# Hostel support workers’ experiences navigating healthcare alongside people experiencing homelessness: a qualitative study in the UK

**DOI:** 10.1136/bmjopen-2024-085949

**Published:** 2024-09-23

**Authors:** Iman Muzafar, Oliver Cunningham

**Affiliations:** 1 Imperial College London, London, UK; 2 GKT School of Medical Education, King's College London, London, UK; 3 Barts and The London School of Medicine and Dentistry, London, UK

**Keywords:** Qualitative Research, Health Equity, Health Services Accessibility, Health Services

## Abstract

**ABSTRACT:**

**Objectives:**

This study aimed to explore how hostel support workers (HSWs) experience navigating healthcare alongside people experiencing homelessness (PEH). PEH experience poor health outcomes, increased mortality and face many barriers when accessing healthcare. HSWs have a dynamic and holistic role, working with PEH to navigate access to health and social care, whilst facilitating independence. HSWs have been described as important in addressing barriers to access and linking healthcare and PEH. However, HSWs’ experiences navigating this role across sectors remains underexplored.

**Design:**

In this qualitative study, semistructured interviews were conducted, and phenomenological thematic analysis was performed.

**Setting:**

Interviews were conducted with participants from 13 hostels across four UK counties.

**Participants:**

17 HSWs were interviewed, with experience in their role ranging from 3 months to over 10 years.

**Results:**

Three themes were identified.

HSWs feeling stigmatised by healthcare staff (HCS), including power inequalities between HSWs and HCS, and the impact of stigma against PEH in healthcare on HSWs.

Working across sectors, including both collaborative and disconnected experiences.

In-reach and its role in linking PEH and healthcare. This included the contrasting subthemes of in-reach as an effective link versus in-reach being an impractical and unsustainable solution.

**Conclusions:**

Cross-sector collaboration has been recognised as an effective way to increase healthcare access and improve outcomes for Inclusion Health Groups, including PEH. This has been further highlighted by the formation of Integrated Care Systems, which strive to bring sectors together to tackle inequalities in outcomes, experience and access. Collaborative relationships between sectors, that is, hostels and healthcare, are vital for increasing healthcare access for PEH. We explored the experiences of HSWs navigating healthcare access for this marginalised population. Recognising and understanding these experiences are the first steps in building collaborative cross-sector relationships to improve healthcare accessibility, experiences and outcomes for PEH.

**STRENGTHS AND LIMITATIONS OF THIS STUDY**

- This study focused on hostel support workers’ experiences, rather than using them as spokespeople for people experiencing homelessness, in order to gain insight into the experiences of this important stakeholder.
- This study focused on front-line hostel support workers, rather than those who do not directly work with people experiencing homelessness.
- Semistructured interviews enabled an in-depth understanding and analysis of the experiences of hostel support workers navigating healthcare, which are transferable across the UK.
- This study was conducted by healthcare students with no funding, meaning that only participants who were able to volunteer their time for free were able to participate.
- Participants needed access to Microsoft Teams to be interviewed, therefore excluding participants without access.

## Introduction

 People experiencing homelessness (PEH) suffer poor health outcomes and increased mortality.^1^,^3^ PEH experience trimorbidity: concurrent substance use disorders, mental health and physical health conditions which contribute to premature deaths.^4^ The social determinants of health, including poverty, education, housing and unemployment, contribute to this increased mortality and morbidity by increasing the likelihood of experiencing homelessness and poor health.^5^ For PEH in the UK, the mean age at death is 45.4 for men and 43.2 for women, compared with the national averages of 79.4 and 83.1, respectively.^6^ One in three of these deaths would have benefited from healthcare input.^7^

PEH are an Inclusion Health Group under National Health Service (NHS) England’s Inclusion Health Framework as they experience social exclusion, poor access to healthcare and multiple overlapping risk factors for poor health, including poverty, violence and complex trauma.^8^ This framework aims to meet the unmet healthcare needs of the most vulnerable and socially excluded individuals in our population.^8^ Despite this effort, PEH’s access to healthcare is riddled with barriers.^9 10^ This includes structural barriers, unmet physiological needs, past negative experiences, loss of trust in the healthcare system and stigmatisation of PEH by healthcare staff (HCS).^2 11 12^ Increasing the flexibility of healthcare services and facilitating supportive relationships between HCS and PEH can partly address these barriers. However, to address the healthcare needs of PEH effectively, research has identified that cross-sector collaboration between sectors, including housing and healthcare, is required.^213^,^16^ Cross-sector collaboration is collaboration in which the strengths of different groups are leveraged to reach a shared goal.^17^ One of these groups are hostel support workers (HSWs), who have been referred to as the link between healthcare and hostels and are therefore fundamental in facilitating access to healthcare for PEH.^18^

The positive and trusting relationship between HSWs and PEH is well supported in the literature,^18^,^20^ although it is not universal among all studies.^21^ This positive relationship between HSWs and PEH has been recognised as ‘an essential step in engaging [PEH] with other services’ (Armstrong et al.^18^ p. 1); however, HSWs’ experiences navigating healthcare has been neglected from research and from the design of interventions aiming to address poor health outcomes and access.

HSWs work within hostels, which are run by various organisations, including local authorities, faith-based and non-governmental organisations.^22^ Hostels aim to facilitate a means towards helping people move out of homelessness.^22^ There are 1185 hostels with over 35 000 bed spaces in England, and over 75 000 individuals use hostels per year.^23^

HSWs typically have a caseload of ‘clients’ who they assist to access financial support, housing, healthcare and more.^24^ HSWs have a multifaceted role. However, they receive limited training and support from health and social care services, resulting in high turnover rates and burnout.^18 25^

PEH continue to experience deteriorating health, increased morbidity and premature mortality whilst accessing homeless accommodation, with 54% of deaths occurring within hostels.^26^ As a result, HSWs often go above and beyond to facilitate access to healthcare services across different specialties.^27^ Navigation involves ‘finding the right care at the right time in the right place’ (Griese et al.^28^ p. 2) and encompasses the literal steps HSWs undertake to ensure that appointments are arranged, attended and followed-up, to guide PEH’s movement through a complex care system.^29^ HSWs also emotionally navigate access to healthcare, assuming a familial and emotionally supportive role.^30^ The experiences of HSWs navigating healthcare alongside PEH remain underexplored.

Armstrong^18^ reported that HSWs felt stigmatised and disbelieved by external services; more evidence is needed to understand and support these findings. HSWs are often viewed as spokespersons for PEH rather than as another key stakeholder who navigate healthcare alongside PEH, with their own experiences to share.^18 25 31^ As a result, we neglect two important stakeholders: HSWs and PEH. We understand little about HSWs’ experiences in their role and neglect to engage directly with PEH, resulting in poorly designed implementations which lack appropriate stakeholder engagement.^32^

This study aimed to develop an understanding of the experiences of HSWs navigating healthcare alongside PEH, considering HSWs to be important stakeholders rather than spokespersons for PEH. This included further exploring the under-researched experiences of stigma against HSWs in healthcare settings.^18^

## Methods

### Research team, reflexivity and positionality

Interviews were conducted by two researchers, one female (IM) and one male (MM), following the Standards for Reporting Qualitative Research framework^33^ (online supplemental material 1 (COREQ)). Two researchers and the participant were present at the semistructured interviews (SSIs): one researcher conducted the interview, while the other took field notes. Field notes were used to gauge general interpersonal dynamics and non-verbal communications within the SSIs. Researchers were Imperial College Business School students, who had undertaken university teaching on qualitative research methods. IM had prior experience conducting qualitative research and interviews. Researchers were healthcare students with clinical experience. Researchers had limited understanding of the experiences of HSWs beyond the existing knowledge in the literature, facilitating an approachable atmosphere during interviews. Rapport was built with participants through email communication prior to conducting SSIs, to encourage participants to feel comfortable sharing experiences. Participants were aware of the researcher’s institution and interests.

### Study design

This study was informed by a phenomenological perspective to better explore the subjective meaning of HSWs’ lived experiences, emotions and interactions as they navigate healthcare alongside PEH. Due to the study’s exploratory nature and to produce new insights, an inductive thematic analysis was adopted to derive themes without being directed by preconceived theoretical constructs. Participants were recruited through purposive criterion sampling (table 1).

**Table 1:** Participant recruitment criteria

Inclusion criteria	Exclusion criteria
HSWs who work directly with PEH	HSWs who do not work directly with PEH (e.g., hostel administrative staff)
English speaking	Non-English speaking
Aged 18 and above	Under the age of 18

HSWshostel support workersPEHpeople experiencing homelessness

Hostel organisations were made aware of the study by email and through a nationally circulated newsletter from the Queens’ Nursing Institute’s Homeless and Inclusion Health Programme. Contact details of the research team were provided, enabling participants to register their interest. 17 HSWs participated from 13 charity and council-funded hostels across four UK counties: Greater London, Norfolk, West Midlands and West Yorkshire. No participant refused or dropped out of the study.

17 SSIs were conducted from 18 April 2023 to 28 April 2023 on Microsoft Teams, lasting 20–45 minutes. Two of these were pilot SSIs to facilitate the reflexive adaptation of the interview guide (online supplemental material 2 (SSI guide)). The interview guide was structured to explore positive experiences before negative experiences, accounting for negativity bias.^34^ Follow-up questions and prompts were included to explore personal experiences.

Each interview was audio-recorded and transcribed verbatim, with no repeat interviews. The data was validated and pseudonymised by the member of the research team taking field notes. To maintain confidentiality, participants were pseudonymised by being assigned codes, for example, HSW1. Following this, audio recordings were destroyed. Transcripts were not sent to participants, as there is little evidence that this enhances the credibility or trustworthiness of qualitative data.^35^ Thematic saturation does not align with the reflexive approach used.^36^

### Data analysis

Reflexive phenomenological thematic analysis was performed through Braun and Clarke’s^36^ revised six-step framework.^36^

This first involved familiarisation with each transcript and forming familiarisation notes. Due to the study’s phenomenological perspective, this stage was prolonged to ensure data immersion, credibility and trustworthiness. IM conducted systematic data coding and management on NVIVO-12 to generate initial codes. Codes were redefined and reviewed in reflective meetings to ensure findings were credible and accurately reflected the data collected. Two researchers (IM and OC), recognising their subjectivity, then analysed the codes to interpret initial themes from the data, with potential subthemes being developed into a thematic map. Subthemes and themes were reviewed and redefined. Exemplar participant quotations were identified and reviewed to illustrate the relevance and consistency of the themes with the data. Participants did not provide feedback on the findings.

### Ethical considerations

Full ethical approval for this study was granted by the Head of Research Governance and Integrity of the Imperial College Research Ethics Committee on 13 April 2023. Reference number: 6 505 789. Written and verbal informed consent was given by participants.

### Patient and public involvement

A Supported Hostels Manager was involved in the study design, interview guide and recruitment of participants. The choice to use the term HCS rather than healthcare professionals in the interview guide and paper, was guided by public involvement and preference. Additionally, the focus on navigating healthcare ‘alongside’, rather than ‘on behalf of’, PEH was guided by HSWs.

## Results

### Themes

**Figure 1:**
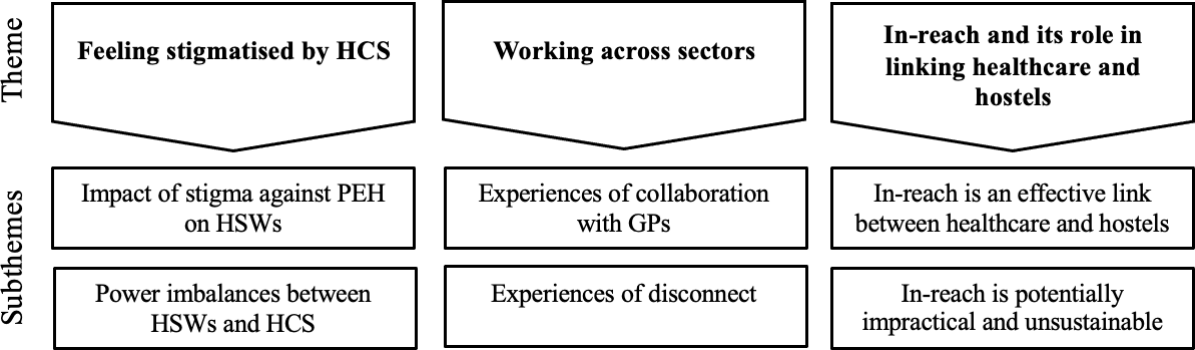
Illustration of themes and subthemes. GP, general practitioner; HCS, healthcare staff; HSWs, hostel support workers; PEH, people experiencing homelessness.

Through phenomenological thematic analysis, we identified three themes and six subthemes, encompassing the experiences of HSWs navigating healthcare alongside PEH (figure 1 and table 2).

**Table 2:** Example of coding tree

Codes	Subthemes	Theme
HSW feeling frustrated by the experiences of PEH in healthcare settingsHSW feeling distressed and upset by directly witnessing stigmatising experiences against PEHHSW feeling distressed and angry by the experiences of PEH in healthcare setting, when HSW are not presentIncreasing workload on HSWs, as a result of the breakdown in relationship between HCS and PEHHSW noticing non-verbal stigma from HCSHSW noticing non-verbal stigma from patientsHSW feeling guilt and blame as they encourage PEH to access healthcare, and then PEH have negative experiences	Impact of stigma against PEH on HSWs	Feeling stigmatised by HCS
HSW feeling undervalued and underappreciated by HCSHSW feeling scrutinised by HCSHSW finding it difficult to speak up against HCSHCS being surprised by HSW knowledge regarding PEH and healthcareHSW feeling blamed by HCS when PEH do not attend appointmentsHSW feeling that they need a job title for HCS to engage with themHSW feeling that HCS view them as lesser, due to assumptions regarding educational attainmentHSW feeling that HCS view them as lesser, due to assumptions regarding job role	Power imbalances between HSWs and HCS	

HCShealthcare staffHSWshostel support workersPEHpeople experiencing homelessness

### Feeling stigmatised by HCS

#### Impact of stigma against PEH on HSWs

PEH experience judgement, discrimination and stigmatisation in healthcare settings. HSWs witness these experiences directly and are also indirectly affected by experiences shared by PEH.

[HSW7 sharing an experience where their client went to smoke and wasn’t let back into hospital] *“He was quite distraught. I asked what had happened and it was security who said that he wasn't meant to be at the hospital so threw him out. They said, ‘you're homeless, you're just trying to be inside where it’s warm, you’re not meant to be here’… He snuck back in and was able to find me, but it was quite distressing for myself and the client.”*

HSWs felt that the wider societal stigmatisation of PEH, in which PEH were perceived as being chaotic, reluctant to engage, and at fault for their housing and health circumstances, resulted in stigmatisation within healthcare settings.

HSWs experienced that HCS preferred to speak to them rather than PEH, with this being the result of “*unconscious biases [where PEH are viewed as chaotic and disengaged], which are just seeping into, not only [HCS] role, but throughout their industry”* (HSW1).

PEH experience direct stigma in healthcare settings, which is spoken and unspoken. Resulting in healthcare avoidance as PEH fear being mistreated:

[HSW14 discussing their experience of observing unspoken stigma, through staring, whilst accompanying a client to an appointment] *“I don't like it because if I can see it and feel it then the person that I'm taking to the appointment can as well. It makes people not want to engage with [healthcare] services because they do feel as if they are looked down upon, that they're a lower class citizen”*.

This stigma has emotional and practical impacts on HSWs. Emotionally, HSWs are left feeling disappointed, frustrated and upset:

[HSW12 discussing how the stigmatisation of PEH impacts them, especially when they observe how PEH are treated differently from themselves] *“It’s quite hard to see, perhaps how, how people could be treating them [PEH] differently when, ultimately, they're a human being and they have the same rights as others. So, I think it can be quite frustrating, it can be quite hard, and perhaps quite upsetting”*.

HSWs invest time in building relationships with PEH, in order to gain trust, and empower PEH to access healthcare. These stigmatising experiences have practical impacts on HSWs’ job and workload, as PEH become increasingly reluctant to use healthcare services due to the ‘*large stigma for some clients about using [NHS services] due to being treated poorly within them in the past’* (HSW12). These stigmatising experiences threaten to undermine HSWs’ relationships with PEH, with HSWs feeling that they must prove their commitment to support PEH in accessing healthcare:

*“Sometimes if I've tried getting an appointment and it’s not worked, the next day I'll be like ‘I'm gonna come wake you up and we're gonna do it together’. Sometimes I sit on hold for half an hour. I don't want them [PEH] to think ‘she’s not bothering’. I show that I’ve tried… It kind of helps and doesn't disrupt the relationship”* (HSW10).

Workload is further increased as HSWs witness the stigmatisation of PEH, and feel a duty to escalate to senior HCS, make complaints and report incidents. This is emotionally burdensome, with HSWs feeling conflicted as they do not want to complain but feel it is the only effective way to be heard.

#### Power imbalance between HSWs and HCS

HSWs felt that they were looked down upon by HCS, who presumed they were inexperienced and underqualified due to assumptions about HSWs’ sector, job role, pay and educational attainment.

*“When it comes to supportive roles, they come in all different shapes and sizes. I think a lot of times they [HCS] assume you're just a carer working for an agency on £7.00 an hour. They [HCS] kind of look downwards and that level of respect is not necessarily there, they [HCS] don’t understand that support workers come with a world of knowledge and experience”* (HSW14).

*“I think there is a bit of a culture of, you know, that they [HCS] don't really see us hostel workers as professionals because they’re healthcare professionals… They [HCS] go to university. You know, a lot of the hostel workers, they've never been to uni, they're not specialists in healthcare”* (HSW11).

This resulted in HSWs’ suggestions being ignored and their expertise being disregarded. Resulting in HCS missing vital pieces of information which could support a holistic approach to PEH’s physical and mental health.

Experiencing these power imbalances did not stop HSWs trying to navigate healthcare access, however, it did result in an ‘us vs them’. HSWs and PEH grow closer together, and become increasingly distant from HCS, threatening the development of collaborative relationships.

[HSW11’s response when asked about how negative experiences with HCS impacts how they navigate access to healthcare] “It’s not gonna impact me because I'll just build a closer relationship with my residents and get the information that I need”.

### Working across sectors

#### Experiences of collaboration with General Practitioners (GPs)

HSWs viewed collaboration across sectors as vital in facilitating healthcare access for PEH, in order to address health inequalities and increase healthcare access and utilisation.

When working across sectors to make hostels and healthcare more connected, there were promising experiences of collaboration shared by HSWs. Collaborative relationships were most commonly experienced with General Practitioners (GPs) and were underpinned by knowledge sharing from HSWs to HCS, alongside knowledge sharing from HCS to HSWs:

*“We [GP and HSWs] have a good relationship. I want to learn from her and vice versa. I think the better we work together, the better we support our clients. So, I think it’s just something that’s happened organically. I always say to her [the GP], I'm excited to learn and I'm happy to have resources sent to me and then vice versa, she'll ask the same”* (HSW7).

Interviewees echoed the importance of sharing knowledge to foster collaborative working relationships and to ensure the needs of PEH are met during GP appointments. The receptiveness of GPs to this information made HSWs feel valued and heard.

#### Experiences of disconnect

Disconnected and disjointed experiences were shared by HSWs and these experiences resulted from poor communication and lack of information sharing.

HSWs particularly struggled in navigating access to routine and emergency dental care. The current NHS dentist shortage means that waiting lists are longer than the length of stay in hostels.

“We've got 12 clients in this hostel, none of them are registered at a dentist currently and the waiting list for dentists is about 4–5 years. With it being emergency accommodation, they will move on in around 3 months” (HSW10).

HSWs also struggled to access and work with mental health services, due to long waiting lists, rejected referrals and prompt discharges from these services:

[HSW 4 discussing challenges and disagreements with mental health services] *“It’s like we see these ladies [PEH] everyday, they live here. We've got a whole history of how they [PEH] are and they [mental health services] just don't listen to us or don't discharge them with the discharge plan and it just seems so chaotic, and it’s like okay, now that’s back in our hands, and we can't really do anything either.”*

HSWs felt that themselves and HCS had opposing priorities and beliefs, meaning that sectors struggle to understand each other and *“can't always come to a level agreement on things”* (HSW4).

HSWs believe in repetition and persistence, which feels incompatible with NHS protocols that discharge patients who do not attend appointments.

[HSW5 discussing re-referring PEH to healthcare services, and the response from HCS] *“They [HCS] say, ‘we offered this last time and they [PEH] didn't arrive, they're just never gonna engage with this’. We [HSWs] have an outlook of consistency and repetitiveness and try, try, try again, whereas health services just cannot do that.”*

Despite these experiences of poor collaboration, disconnect and opposing beliefs, cross-sector collaboration remained highly important to address the healthcare needs of PEH:

*“There’s a certain amount of prejudice towards homelessness and towards people that live within hostel accommodation, and oftentimes the people that require that do have quite chaotic lives. But they will only become more chaotic without the support of the external agencies, and health does come into that. I think it’s a shame, really, that there’s this disconnect between the sort of support services and health services”* (HSW1).

General Data Protection Regulation (GDPR) was frequently cited as a regulatory barrier to information sharing, and some HSWs found that confidentiality releases and consent forms helped overcome this barrier. HSWs felt that GDPR may be used as an excuse by HCS to avoid information sharing. As a result, HSWs become reluctant to share information with HCS.

*“Resistance around GDPR, that’s becoming a big problem. I don't know if that’s an excuse… Since this new law around GDPR, I’ve found it more difficult to do my job and get the information I need to keep people safe”* (HSW11).

### In-reach and its role in linking healthcare and hostels

#### In-reach is an effective link between healthcare and hostels

In-reach is when HCS bring their expertise and services into hostels, directly to PEH. We found that the most common in-reach services were nurse-led programmes, sexual health screening and immunsations (table 3).

**Table 3:** Hostel demographics

Characteristic of hostel	Number of hostels (n=13)
**Location**	
Greater London	9
West Yorkshire	2
Norfolk	1
West Midlands	1
**Hostel funding**	
Charity	10
Council	3
**Residents level of care needs**	
High	5
Medium	4
Low	1
Prefer not to say	3
**In-reach services availabe**	
No	2
Yes	11
Nurse-led general health clinics	10
Sexual health	5
Immunisations	5
General Practitioner (GP)	4
Drug and addiction	2
Podiatry	2
Mental health	1
Respiratory health	1
Clinical psychologists	1
**Residentialcapacity**	
<20	3
21–40	3
41–60	4
61–80	1
Prefer not to say	2
**Length of stay**	
Up to 2 years	9
Up to 5 years	1
Prefer not to say	3

HSWs viewed themselves as a link between healthcare and hostels in some ways, but felt that in-reach offered a more effective link due to the closer association between in-reach and external healthcare.

HSWs working at hostels with in-reach felt that this was able to effectively link healthcare and hostels. This was due, in part, to logistical reasons, including a shared electronic infrastructure between in-reach and external healthcare teams:

*“We'll go through the in-reach services that we work with, and because they have access to like the hospital system, they can pass on anything that we do need”* (HSW9).

The effectiveness of in-reach largely came down to familiarity. The same HCS would often come to the hostel, with this consistency being compatible and complementary to HSWs’ organisational beliefs surrounding repetition. Relationships between in-reach HCS, HSWs and PEH developed organically.

*“When she [in-reach HCS] comes in, she will knock on their [PEH] door and can give them time slots that they can come down or she tells them ‘I'm here from this time till that time so come and see me’. At least they [PEH] know that, even if they can't get an appointment at a doctor’s surgery, this person will be here to see them at that time”* (HSW2).

#### In-reach is a potentially impractical and unsustainable solution

The sustainability and practicality of in-reach was questioned. HSWs explored whether in-reach could provide the same standard of care as secondary care hospital settings, as fewer resources, equipment and facilities were available. Although in-reach was viewed as better than no care, it was not perceived as the ultimate solution to ensure PEH access healthcare:

*“In-reach can only go so far, if you bring stuff into the hostel, that hasn't got all the hospital facilities and everything, so it’s brought to light the difficulty of and the importance of perhaps finding solutions and finding those ways to bridge that gap”* (HSW12).

Additionally, in-reach did not provide a self-sufficient solution to increasing access to healthcare for PEH. HSWs still had a significant workload to ensure in-reach was utilised, by preparing PEH for in-reach by “*knocking on doors, giving reminders, having posters up”* (HSW7).

## Discussion

This paper explored HSWs’ experiences navigating healthcare alongside PEH. We identified three overarching themes summarising these experiences: feeling stigmatised by HCS, working across sectors and the role of in-reach in linking healthcare and hostels. The stigmatisation of PEH in healthcare is well established and results in reduced healthcare access and utilisation.^12^ This study explored the impact of this on HSWs, who witness these stigmatising interactions and, as a result, experience frustration, distress and upset. Previous research has identified that HSWs can also feel stigmatised by healthcare services,^18^ and this study supports these findings and provides a more nuanced understanding of this stigmatisation. This stigmatisation results from HSWs having roles that are poorly understood by HCS, with HSWs feeling that HCS presume they are ‘*under-qualified’* or ‘*unprofessional’*. The educational attainment of HSWs interviewed was highly variable, with the highest educational qualifications ranging from General Certificate of Secondary Education (GCSE) to Masters (table 4). A workforce survey of HSWs would help ascertain whether our interviewees reflect the broader profession. This study has potential limitations. Interviews were conducted via Microsoft Teams, therefore excluding HSWs without access. Interviewees were not reimbursed, so only those able to volunteer their time were interviewed, possibly resulting in self-selection bias. Healthcare students conducted the interviews, potentially resulting in a response bias in which HSWs felt unable to share negative experiences.

**Table 4:** Participant demographics

Characteristic	Participants/HSWs (n=17)
**Jobtitle**	
Support worker	6
Team leader	5
Specialist health lead	2
Sport manager	1
Deputy manager	1
Mental health lead	1
Supported pathways officer	1
**Age**	
25–34	0
35–44	9
45–54	1
55–64	0
65+	1
Prefer not to say	6
**Ethnicity**	
White British	8
Black Caribbean	2
Black African	1
Latin American	1
White Other	1
Prefer not to say	4
**Gender**	
Female	8
Male	5
Prefer not to say	4
**Length of experience workingwith PEH**	
<1 year	1
Up to 2 years	7
Up to 3 years	3
Up to 5 years	2
Up to 10 years	2
>10 years	2
**Educational attainment(FHEQ Level)**	
2 (GCSEs or eq.)	3
3 (A-levels or eq.)	1
4 (Certificate of higher education or eq.)	0
5 (Foundation degree or eq.)	0
6 (Bachelor’s degree or eq.)	6
7 (Master’s degree or eq.)	3
Prefer not to say	4

FHEQFramework for Higher Education Qualifications for England, Wales and Northern IrelandGCSEGeneral Certificate of Secondary EducationHSWshostel support workersPEHpeople experiencing homelessness

A cross-sector approach has been identified as being fundamental in supporting the complex needs of PEH,^37 38^ driving the creation of shared goals across sectors to improve patient care and outcomes and reducing power imbalances between sectors.^39^ The opposing organisational culture between hostels and healthcare is a barrier to cross-sector collaboration. However, collaborative experiences with GPs demonstrate potential solutions to address these challenges. These experiences were underpinned by knowledge transfer from HCS to HSWs, as well as from HSWs to HCS. The importance of HCS receptiveness to HSWs’ knowledge was a novel finding, and HSWs felt that this facilitated working relationships across sectors. This study highlighted experiences of disconnect, disjointedness and lack of collaboration between sectors, with HSWs particularly struggling to navigate access to dental care and mental health services. While other studies have discussed the lack of responsiveness and information sharing from healthcare,^18 26^ this study went further and explored how sectors can struggle to understand each other and their respective priorities and, as a result, fail to effectively collaborate and reach agreements in the interest of PEH. GDPR, as a barrier to collaboration across sectors, has been identified in previous research.^18^ However, this paper also recognised practical solutions to address this issue, including consent forms and confidentiality releases.

Ensuring collaboration and bridging gaps between healthcare, social care, local authority services and the voluntary, community and social enterprise sector is fundamental to improving health outcomes and reducing inequalities.^40^ The importance of this has been brought to the forefront of the NHS through the formation of Integrated Care Systems, which aim to tackle inequalities in outcome, experience and access.^40^ Integrated Care Systems depend on the establishment and strengthening of collaborative relationships between sectors.^40^ For PEH, the experiences of disconnect and poor collaboration between HSWs and HCS threaten the likelihood of successful collaboration between hostels and healthcare providers.

In-reach services fostered positive experiences for HSWs. Despite the literature viewing HSWs as the link between PEH and healthcare,^18^ this paper identified that HSWs perceive in-reach to be the more effective intermediary, indicating that initiatives focused on training and upskilling HSWs may be less effective at addressing the poorer health outcomes facing PEH.^32^ This paper also raises questions regarding the definition of in-reach; whilst papers have classified in-reach as two half-days per month,^32^ this paper identified that HSWs define in-reach as a weekly initiative. HSWs questioned the sustainability and practicality of in-reach, as HSWs felt there were situations in which external healthcare was needed due to the availability of specialised equipment and facilities. This paper identified that while in-reach is effective in facilitating access to healthcare for PEH and provides great support to HSWs, it cannot eliminate the need for external healthcare, which remains vital in improving health outcomes for PEH. The continuing development of solutions to address access to external healthcare should therefore be a priority when planning future service provisions and undertaking further research.

## Conclusion

This paper explored the experiences of HSWs navigating healthcare alongside PEH, from the perspective of HSWs themselves. Negative experiences which hinder collaboration were shared, alongside potential solutions to building more positive and collaborative relationships. Recognising and understanding these experiences are the first steps in building collaborative relationships across sectors to improve accessibility, experiences and outcomes for PEH.

## Implications for research and clinical practice

Stakeholder engagement is vital in designing relevant and useful strategies to address inequalities in access to healthcare for PEH. Neglecting stakeholders threatens the success of inclusion health initiatives for PEH. This research focuses on HSWs as individuals with their own experiences to share rather than as spokespeople for PEH.

It is evident that the relationship across sectors is fragmented; HSWs feel stigmatised and disconnected from HCS and healthcare. Cross-sector collaboration is underpinned by trust, stakeholder engagement throughout and a dynamic approach to role definitions.^41^ The practicalities of collaboration, in the context of hostels and healthcare, need to be further studied, with Integrated Care Systems offering an opportunity to understand how this develops over time.

Whether in-reach compromises the quality of healthcare received by PEH in comparison to care provided by external healthcare needs to be further explored.

There are significant challenges surrounding information sharing and GDPR compliance. In clinical practice, the use of consent forms and confidentiality releases by hostels may mitigate these challenges, providing these documents are recognised by the healthcare system.

## supplementary material

10.1136/bmjopen-2024-085949 online supplemental file 1

10.1136/bmjopen-2024-085949 online supplemental file 2

## Data Availability

Data are available upon reasonable request.

## References

[1] Fazel S, Geddes JR, Kushel M (2014). The health of homeless people in high-income countries: descriptive epidemiology, health consequences, and clinical and policy recommendations. The Lancet.

[2] McNeill S, O’Donovan D, Hart N (2022). Access to healthcare for people experiencing homelessness in the UK and Ireland: a scoping review. BMC Health Serv Res.

[3] Nadicksbernd JJ, Nguyen T, Jackson T (2023). Health and care needs of hospitalised people experiencing homelessness: an inpatient audit. *Clin Med (Lond*).

[4] Hewett N, Halligan A (2010). Homelessness is a healthcare issue. J R Soc Med.

[5] Baldry E, Dowse L, McCausland R (2012). Lifecourse *institutional costs of homelessness for vulnerable groups: department of families, housing, community services and indigenous a*ffairs.

[6] Butt A, John E (2019). Deaths of homeless people in England and Wales: 2018, deaths of homeless people in England and Wales. https://www.ons.gov.uk/peoplepopulationandcommunity/birthsdeathsandmarriages/deaths/bulletins/deathsofhomelesspeopleinenglandandwales/2018.

[7] Aldridge RW, Menezes D, Lewer D (2019). Causes of death among homeless people: a population-based cross-sectional study of linked hospitalisation and mortality data in England. Wellcome Open Res.

[8] NHS England (2023). https://www.england.nhs.uk/long-read/a-national-framework-for-nhs-action-on-inclusion-health/.

[9] Schanzer B, Dominguez B, Shrout PE (2007). Homelessness, health status, and health care use. Am J Public Health.

[10] Bedmar MA, Bennasar-Veny M, Artigas-Lelong B (2022). Health and access to healthcare in homeless people. Medicine (Baltimore).

[11] Omerov P, Craftman ÅG, Mattsson E (2020). Homeless persons’ experiences of health‐ and social care: a systematic integrative review. *Health Soc Care Community*.

[12] Reilly J, Ho I, Williamson A (2022). A systematic review of the effect of stigma on the health of people experiencing homelessness. *Health Social Care Comm*.

[13] Crane M, Joly L, Daly BJ (2023). Integration, effectiveness and costs of different models of primary health care provision for people who are homeless: an evaluation study. *Health Soc Care Deliv Res*.

[14] Corrigan P, Pickett S, Kraus D (2015). Community-based participatory research examining the health care needs of African Americans who are homeless with mental illness. J Health Care Poor Underserved.

[15] Shulman C, Hudson BF, Low J (2018). End-of-life care for homeless people: a qualitative analysis exploring the challenges to access and provision of palliative care. Palliat Med.

[16] van Laere IR, de Wit MA, Klazinga NS (2009). Pathways into homelessness: recently homeless adults problems and service use before and after becoming homeless in Amsterdam. BMC Public Health.

[17] (2022). Working in Partnership with People and Communities: Statutory Guidance. https://www.england.nhs.uk/long-read/working-in-partnership-with-people-and-communities-statutory-guidance/.

[18] Armstrong M, Shulman C, Hudson B (2021). Barriers and facilitators to accessing health and social care services for people living in homeless hostels: a qualitative study of the experiences of hostel staff and residents in UK hostels. BMJ Open.

[19] Hall T (2006). Out of work and house and home: contested youth in an English homeless hostel. Ethnos.

[20] McGrath L, Pistrang N (2007). Policeman or friend? Dilemmas in working with homeless young people in the United Kingdom. J Soc Issues.

[21] Neale J, Stevenson C (2015). Social and recovery capital amongst homeless hostel residents who use drugs and alcohol. Int J Drug Policy.

[22] Busch-Geertsema V, Sahlin I (2007). The role of hostels and temporary accommodation. Eur J Homelessness.

[23] Reach T (2017). Statistics Homelessness Facts and Figures - thamesreach.org.uk.

[24] Manthorpe J, Samsi K, Joly L (2019). Service provision for older homeless people with memory problems: a mixed-methods study. *Health Serv Deliv Res*.

[25] Jagpal P, Saunders K, Plahe G (2020). Research priorities in healthcare of persons experiencing homelessness: outcomes of a national multi-disciplinary stakeholder discussion in the United Kingdom. Int J Equity Health.

[26] Shulman C, Nadicksbernd JJ, Nguyen T (2023). People living in homeless hostels: a survey of health and care needs. *Clin Med (Lond*).

[27] Pendyal A, Rosenthal MS, Spatz ES (2021). “When you’re homeless, they look down on you”: a qualitative, community-based study of homeless individuals with heart failure. H L.

[28] Griese L, Berens E-M, Nowak P (2020). Challenges in navigating the health care system: development of an instrument measuring navigation health literacy. Int J Environ Res Public Health.

[29] Freeman HP, Rodriguez RL (2011). History and principles of patient navigation. Cancer.

[30] de Veer AJE, Stringer B, van Meijel B (2018). Access to palliative care for homeless people: complex lives, complex care. BMC Palliat Care.

[31] Howells K, Burrows M, Amp M (2021). Exploring the experiences of changes to support access to primary health care services and the impact on the quality and safety of care for homeless people during the covid-19 pandemic: a study protocol for a qualitative mixed methods approach. Int J Equity Health.

[32] Armstrong M, Shulman C, Hudson B (2021). The benefits and challenges of embedding specialist palliative care teams within homeless hostels to enhance support and learning: perspectives from palliative care teams and hostel staff. Palliat Med.

[33] Tong A, Sainsbury P, Craig J (2007). Consolidated Criteria for Reporting Qualitative Research (COREQ): a 32-item checklist for interviews and focus groups. Int J Qual Health Care.

[34] Corns J (2018). Rethinking the negativity bias. Rev Philos Psychol.

[35] Thomas DR (2017). Feedback from research participants: are member checks useful in qualitative research?. Qual Res Psychol.

[36] Braun V, Clarke V (2020). One size fits all? What counts as quality practice in (reflexive) thematic analysis. Qual Res Psychol.

[37] Batchelor P, Kingsland J (2020). Improving the health of the homeless and how to achieve it within the new NHS architecture. Int J Environ Res Public Health.

[38] Mosley JE (2021). Cross-sector collaboration to improve homeless services: addressing capacity, innovation, and equity challenges. Ann Am Acad Pol Soc Sci.

[39] Babiker A, El Husseini M, Al Nemri A (2014). Health care professional development: working as a team to improve patient care. Sudan J Paediatr.

[40] Charles A (2022). Integrated Care Systems Explained. https://www.kingsfund.org.uk/publications/integrated-care-systems-explained.

[41] Vooren NJE van, Janssen LMS, Drewes HW (2023). How to collaborate for health throughout the project timeline - a longitudinal study reflecting on implemented strategies in three projects for a healthy living environment. BMC Public Health.

