# Supplementary material for: Hostel support workers’ experiences navigating healthcare alongside people experiencing homelessness: a qualitative study in the UK
Source: BMJ Open. 2024 Sep 23;14(9):e085949. doi: 10.1136/bmjopen-2024-085949 (PMC11418567; doi:10.1136/bmjopen-2024-085949)
Supplement: online supplemental file 1 [file bmjopen-14-9-s001.pdf]

**No. Item**	**Guide questions/description**	**Reported on Page #**
**Domain 1: Research team and reflexivity**		
Personal Characteristics		
1. Interviewer/facilitator	Which author/s conducted the interview or focus group?	3
2. Credentials	What were the researcher's credentials? E.g. PhD, MD	3
3. Occupation	What was their occupation at the time of the study?	3
4. Gender	Was the researcher male or female?	3
5. Experience and training	What experience or training did the researcher have?	3
Relationship with participants		
6. Relationship established	Was a relationship established prior to study commencement?	3
7. Participant knowledge of the interviewer	What did the participants know about the researcher? e.g. personal goals, reasons for doing the research	3
8. Interviewer characteristics	What characteristics were reported about the inter viewer/facilitator? e.g. Bias, assumptions, reasons and interests in the research topic	3
**Domain 2: study design**		
Theoretical framework		
9. Methodological orientation and Theory	What methodological orientation was stated to underpin the study? e.g. grounded theory, discourse analysis, ethnography, phenomenology, content analysis	3
Participant selection		

10. Sampling	How were participants selected? e.g. purposive, convenience, consecutive, snowball	3
11. Method of approach	How were participants approached? e.g. face-to-face, telephone, mail, email	4
12. Sample size	How many participants were in the study?	4
13. Non-participation	How many people refused to participate or dropped out? Reasons?	4
Setting		
14. Setting of data collection	Where was the data collected? e.g. home, clinic, workplace	4<br>.
15. Presence of non-participants	Was anyone else present besides the participants and researchers?	3
16. Description of sample	What are the important characteristics of the sample? e.g. demographic data, date	5-7 (Table 2, Table 3)
Data collection		
17. Interview guide	Were questions, prompts, guides provided by the authors? Was it pilot tested?	4
18. Repeat interviews	Were repeat interviews carried out? If yes, how many?	4
19. Audio/visual recording	Did the research use audio or visual recording to collect the data?	4
20. Field notes	Were field notes made during and/or after the inter view or focus group?	4
21. Duration	What was the duration of the inter views or focus group?	4
22. Data saturation	Was data saturation discussed?	4
23. Transcripts returned	Were transcripts returned to participants for comment and/or correction?	4
**Domain 3: analysis and findings**		
Data analysis		
24. Number of data coders	How many data coders coded the data?	4

25. Description of the coding tree	Did authors provide a description of the coding tree?	7
26. Derivation of themes	Were themes identified in advance or derived from the data?	4
27. Software	What software, if applicable, was used to manage the data?	4
28. Participant checking	Did participants provide feedback on the findings?	4
Reporting		
29. Quotations presented	Were participant quotations presented to illustrate the themes/findings? Was each quotation identified? e.g. participant number	7-11
30. Data and findings consistent	Was there consistency between the data presented and the findings?	7-11
31. Clarity of major themes	Were major themes clearly presented in the findings?	7-11
32. Clarity of minor themes	Is there a description of diverse cases or discussion of minor themes?	7-11
