# Supplementary material for: Hostel support workers’ experiences navigating healthcare alongside people experiencing homelessness: a qualitative study in the UK
Source: BMJ Open. 2024 Sep 23;14(9):e085949. doi: 10.1136/bmjopen-2024-085949 (PMC11418567; doi:10.1136/bmjopen-2024-085949)
Supplement: online supplemental file 2 [file bmjopen-14-9-s002.pdf]

Interview Guide

Version 2.2 19/04/2023

Reflexive adaptations from the two pilot semi-structured interviews are underlined

We are doing this study to learn about your experiences delivering, coordinating, and accessing healthcare on behalf of People Experiencing Homelessness. Some questions may lead you to answer in short or in one word; but please keep in mind there are no right or wrong answers. We are interested in learning about your experiences and thoughts so please try to elaborate on this as much as you can.

Background:

1. Could you briefly tell me about your current role and how long you have worked with People Experiencing Homelessness?

Healthcare access broadly: Thank you for answering those questions, now we will discuss your experiences accessing healthcare from external services, so not in-reach, more broadly.

2. a) Could you tell me about a time when you were able to easily access healthcare for a service user?
   b. Can you tell me about a time when it was difficult to access healthcare for a service user?
   c. Overall, do you find accessing healthcare to be straightforward or challenging?
   d. Do you feel that these experiences have impacted your likelihood to access healthcare services on behalf of your service users in the future?

Experiences with healthcare staff: Now we will ask about your personal experiences with healthcare staff, this could be nurses, doctors such as GPs or anyone else you can think of and the impact this may have had on you and how these experiences made you feel:

3. When engaging with healthcare staff do you feel listened to? *If yes, then why? If no, then why not?*
4. When engaging with healthcare staff do you feel they are open to share information with you? *If yes, then why? If no, then why not?*
   i) *If no:* Are these experiences you have regarding information sharing consistent, or have there been times when you have found that healthcare staff were open to sharing information with you? *If yes, why do you think that was?*
5. a) From your experiences do you feel that healthcare staff understand and appreciate this knowledge and expertise, in terms of your ability to communicate with and build trust with People Experiencing Homelessness, that you have? *If yes, then why. If no, then why not?*
   b. Do you feel able to share your knowledge and expertise with healthcare staff? *If yes, then why. If no, then why not?*
6. Have you ever felt like the healthcare you have delivered was scrutinised by healthcare staff? *If yes, was there anything in particular that was said or done that made you feel this*

way?

7. a) Could you tell me about the last positive experience you had with healthcare staff in your role?

   b. Could you tell me about the last negative experience you had with healthcare staff in your role?

   c. Overall, how have your experiences with healthcare staff been in your role?

   d. As a result of these experiences, could you tell me about how you feel towards healthcare staff?

In-reach: Finally, just a few questions about whether your hostel has any in-reach healthcare services. *(in-reach includes GP/nurse, MH, drug and alcohol)*

8. If your hostel has these, could you tell us briefly about the in-reach healthcare services in place. *(how regularly do they come in, is it the same person, what does this involve?)*

9. a) Could you tell me about some similarities in your experiences with the in-reach healthcare staff, compared to with other healthcare staff?

   b) Could you tell me about your experiences with your in-reach healthcare staff, compared to with other healthcare staff?
