# Supplementary material for: Hostel support workers’ experiences navigating healthcare alongside people experiencing homelessness: a qualitative study in the UK
Source: BMJ Open. 2024 Sep 23;14(9):e085949. doi: 10.1136/bmjopen-2024-085949 (PMC11418567; doi:10.1136/bmjopen-2024-085949)
Supplement: Supplementary file 3 [file bmjopen-2024-085949.reviewer_comments.pdf]

PEER REVIEW HISTORY

BMJ Open publishes all reviews undertaken for accepted manuscripts. Reviewers are asked to complete a checklist review form and are provided with free text boxes to elaborate on their assessment. These free text comments are reproduced below.

ARTICLE DETAILS

**TITLE (PROVISIONAL)**	Hostel support workers' experiences navigating healthcare alongside people experiencing homelessness: a qualitative study in the UK
**AUTHORS**	Muzafar, Iman; Cunningham, Oliver

VERSION 1 - REVIEW

**REVIEWER NAME**	Schiffler, Tobias
**REVIEWER AFFILIATION**	Medical University of Vienna
**REVIEWER CONFLICT OF INTEREST**	None to declare.
**DATE REVIEW RETURNED**	27-Mar-2024

**GENERAL COMMENTS**	This paper presents a compelling and valuable study, exploring the crucial role of hostel support workers (HSWs) in navigating healthcare access for people experiencing homelessness (PEH). It is evident that the research addresses a significant gap in understanding the dynamics between HSWs, healthcare staff, and organizational cultures, from the point of view of HSWs, thereby offering insights into potential improvements in healthcare accessibility for PEH. The methodological approach, utilizing semi-structured interviews and phenomenological thematic analysis, is robust and appropriate for the study's objectives. Overall, the study is an important contribution to the field, with the potential to inform and inspire meaningful changes in how healthcare services are provided to one of the most vulnerable populations. Nevertheless, I have some comments to the authors that they should consider and address.<br><br>ABSTRACT<br>Page 2, line 29:<br>Maybe a sentence describing the background and work of HSWs would contribute to a better understanding of the objective, as this concept is not common in other countries.<br><br>Page 2, line 37:<br>More information on the characteristics of participants included in this study would be beneficial here.<br><br>ARTICLE SUMMARY - STRENGTHS AND LIMITATIONS OF THIS STUDY<br>Page 3, line 13:<br>Please spell the acronym "SSI" (semi-structured interviews) in full as not all readers will know it.

	Page 3, line 20:<br>The authors write about "reduced representativeness of the study's findings". What about transferability of the results to other settings and contexts?<br><br>INTRODUCTION<br>Page 3, line 24:<br>Please elaborate more on the causes (and causes of the causes, i.e. social determinants of health) that make PEH face a higher prevalence of adverse health outcomes and higher mortality.<br><br>Page 3, line 29:<br>How are Inclusion Health Groups defined according to the cited NHS framework?<br><br>Page 3, line 35-37:<br>"Although increasing the flexibility of services and supportive relationships between healthcare and PEH can partly address some of these barriers, in order to address the needs of PEH effectively, research has identified that cross-sector collaboration is required [2, 11, 12, 13, 14].." - Do you mean "healthcare staff" here? Also, please rewrite this multi-clause sentence more "to the point".<br><br>Page 3, line 51:<br>Could you please also describe what the background of HSWs is? Do they receive training prior to working with PEH? Are they peers? It would also be interesting to know if the professional background of HSW is a factor that has an influence on how they are perceived by external services.<br><br>METHODS<br>Research Team and Reflexivity<br>Page 4, line 30:<br>Researcher's name (IM) is not mentioned in the paper but should be in terms of transparency.<br><br>Page 4, line 31-32:<br>In this section, I would highly recommend to add a statement on researcher positionality regarding the two interviewers. What role did their positionality play during the interviews and on what participants said? Did it influence the interactions between interviewers and respondents?<br><br>Page 4, line 33:<br>In what way did the field notes feed into the analysis? What were the researchers' observations (e.g., in terms of interpersonal dynamics between interviewers and respondents) during the interviews?<br><br>Study design<br>Page 5, line 9:<br>How and why was the initial interview guide adapted after the two pretest interviews? Please also indicate this within the interview guide in Appendix 2.<br><br>Page 5, line 17:<br>Qualitative data is hardly anonymizable and the process described in this section is, in fact, referred to as pseudonymization (i.e. assigning individual codes to interviews instead of participant

	names). If data were actually anonymized, please explain further what concrete methods were applied in order to achieve this.<br><br>Page 5, line 20-21:<br>Please elaborate on the methods used to detect the degree of thematic saturation (of upwards of 90%)?<br><br>RESULTS<br>Themes:<br>A general comment regarding the results section: While the findings of this study are very interesting, I believe there is room for a more in-depth exploration. The authors occasionally present single words from participants as "quotes," which might limit the richness of the analysis as providing full sentences instead could offer crucial context, enhancing readers' comprehension of the participants' perspectives. Overall, this section contains very little information and should definitely be extended. This goes especially for the subtheme "In-reach is a potentially impractical and unsustainable solution".<br><br>Page 8, line 33:<br>Has participants' ethnicity played a role in terms of the degree of stigmatization they experienced by external services?<br><br>Page 8, line 36-48:<br>The quotes "underqualified" (HSW9) and “I think there is a bit of a culture of, you know, that [HCS] don't really see us hostel workers as professionals because they’re healthcare professionals. [HCS] go to university. You know, a lot of the hostel workers, they've never been to uni, they're not specialists in healthcare” (HSW11) make me particularly curious on what the qualifications and backgrounds of HSW are (see comment above).<br><br>Page 9, line 19:<br>What are the (potential) reasons for dental care and mental health services being more difficult to navigate for HSWs?<br><br>DISCUSSION<br>Page 10, line 29:<br>Again, it would be beneficial to explain more about the background and qualification of HSWs and how these influence the perception of this group among other professionals.<br><br>Page 10, line 40:<br>Why is it particularly hard for HSWs to navigate access to dental care and mental health services?<br><br>Page 10, line 53:<br>Please spell out the acronym ICS (Integrated Care Systems) as it is only used once throughout the manuscript.<br><br>Page 11, line 26:<br>I urge the authors to add a concluding recommendation on how specific policies currently in force would have to be adapted in order to improve HSWs' healthcare navigation alongside PEH, particularly in terms of the issues that participants raised concerning GDPR.

**REVIEWER NAME**	Patel, R.
**REVIEWER AFFILIATION**	Research Institute for Health and Wellbeing, Coventry University

**REVIEWER CONFLICT OF INTEREST**	no competing interests
**DATE REVIEW RETURNED**	27-Mar-2024

VERSION 1 – AUTHOR RESPONSE

Reviewer 1 comments:

This paper presents a compelling and valuable study, exploring the crucial role of hostel support workers (HSWs) in navigating healthcare access for people experiencing homelessness (PEH). It is evident that the research addresses a significant gap in understanding the dynamics between HSWs, healthcare staff, and organizational cultures, from the point of view of HSWs, thereby offering insights into potential improvements in healthcare accessibility for PEH. The methodological approach, utilizing semi-structured interviews and phenomenological thematic analysis, is robust and appropriate for the study's objectives. Overall, the study is an important contribution to the field, with the potential to inform and inspire meaningful changes in how healthcare services are provided to one of the most vulnerable populations. Nevertheless, I have some comments to the authors that they should consider and address.

Author response: Thank you for spending time reviewing our paper. We appreciate your kind words and have improved our paper to incorporate your comments and feedback.

Abstract:

Maybe a sentence describing the background and work of HSWs would contribute to a better understanding of the objective, as this concept is not common in other countries.

Author response: We agree and have addressed this within the 300 word constraints of the abstract. “HSWs have a dynamic and holistic role, working with PEH to navigate access to health and social care, whilst facilitating independence” on page 1.

More information on the characteristics of participants included in this study would be beneficial here.

Author response: We have included more background on participants in terms of their experience working in hostels to address this comment. The abstract also contain information on hostel characteristics. Further information on participant characteristics are contained within the full text, to ensure the abstract remains below the 300 word limit.

Article Summary - Strengths and limitations of this study

Please spell the acronym "SSI" (semi-structured interviews) in full as not all readers will know it.

Author response: Thank you for pointing this out, we have now changed this to semi-structured interviews rather than SSI (Page 2).

The authors write about "reduced representativeness of the study's findings". What about transferability of the results to other settings and contexts?

Author response: We have now clarified that our findings are transferable within the UK in the strengths and limitation section (Page 2). This is supported further by our discussion which highlights that our findings are transferable and reflective of findings in the literature (Page 11 and 12).

Introduction:

Please elaborate more on the causes (and causes of the causes, i.e. social determinants of health) that make PEH face a higher prevalence of adverse health outcomes and higher mortality.

Author response: We have elaborated on the increased mortality and morbidity experienced by PEH, including information regarding tri-morbidity and the social determinants of health, on page 2.

How are Inclusion Health Groups defined according to the cited NHS framework?

Author response: This definition is now included in the introduction on page 2.

“PEH are an Inclusion Health Group under NHS England’s Inclusion Health Framework as they experience social exclusion, poor access to healthcare and multiple overlapping risk factors for poor health, including poverty, violence and complex trauma” (Page 2)

"Although increasing the flexibility of services and supportive relationships between healthcare and PEH can partly address some of these barriers, in order to address the needs of PEH effectively, research has identified that cross-sector collaboration is required [2, 11, 12, 13, 14].." - Do you mean "healthcare staff" here? Also, please rewrite this multi-clause sentence more "to the point".

Author response: Thank you for pointing this out. We did mean HCS, and have now corrected this. We have also re-written this sentence in line with your feedback, to make it more succinct and clear (page 2).

Could you please also describe what the background of HSWs is? Do they receive training prior to working with PEH? Are they peers? It would also be interesting to know if the professional background of HSW is a factor that has an influence on how they are perceived by external services.

Author response: We have now provided more information on what is known about the background of HSW on page 3, this included adding source 24. “HSWs typically have a caseload of ‘clients’ who they assist to access financial support, housing, healthcare and more [24]. HSWs have a multifaceted role, however they receive limited training and support from health and social care services, resulting in high turnover rates and burnout [18, 31]” (Page 3).

METHODS

Research Team and Reflexivity

Researcher's name (IM) is not mentioned in the paper but should be in terms of transparency.

Author response: The full name is now mentioned in the paper, under author contribution on page 13.

In this section, I would highly recommend to add a statement on researcher positionality regarding the two interviewers. What role did their positionality play during the interviews and on what participants said? Did it influence the interactions between interviewers and respondents?

Author response: Positionality statement has been included in Page 3. This includes researchers experiences as healthcare student with experiences on clinical placement.

“Researchers were healthcare students with clinical placement experience, who had limited understanding of the experiences of HSWs beyond the existing knowledge in the literature, facilitating an approachable atmosphere during interviews.” (Page 3)

In what way did the field notes feed into the analysis? What were the researchers' observations (e.g., in terms of interpersonal dynamics between interviewers and respondents) during the interviews?

Author response: Use of field notes has been clarified under methods on page 3. This remains concise in order to keep the word count lower where possible.

Study design

How and why was the initial interview guide adapted after the two pretest interviews? Please also indicate this within the interview guide in Appendix 2.

Author response: The interview guide in the supplementary material has been amended to show adaptations following pilot interviews. This has been done to ensure this important comment is addressed, without lengthening the paper or contributing significantly to the word count. Additionally, we feel it is more understandable to read the edited sections within the interview guide itself.

Qualitative data is hardly anonymizable and the process described in this section is, in fact, referred to as pseudonymization (i.e. assigning individual codes to interviews instead of participant names). If data were actually anonymized, please explain further what concrete methods were applied in order to achieve this.

Author response: Thank you for highlighting this. Our data was pseudonymised not anonymised, and we have now made this clear in the paper (Page 4).

Please elaborate on the methods used to detect the degree of thematic saturation (of upwards of 90%)?

Author response: We have now removed this statement from our paper, following your comment and reviewing the 2020 revised paper and recommendations by Braun and Clarke. We have realised that the aim of thematic saturation is not applicable and is not consistent with reflexive TA. Our previous interpretation of this was that at least 12 interviews are needed to discern thematic saturation, and this is based on a base sample of interviews, running length of the interviews and then when themes start repeating themselves. We apologise for any confusion caused (Page 4).

Results:

Themes:

A general comment regarding the results section: While the findings of this study are very interesting, I believe there is room for a more in-depth exploration. The authors occasionally present single words from participants as "quotes," which might limit the richness of the analysis as providing full sentences instead could offer crucial context, enhancing readers' comprehension of the participants' perspectives. Overall, this section contains very little information and should definitely be extended. This goes especially for the subtheme "In-reach is a potentially impractical and unsustainable solution".

Author response: Thank you for this constructive feedback. We have included all of your comments. We have removed one word quotes from the results and have re-written and extended this section so it contains more information. This has occurred for all themes and subthemes, including "In-reach is a potentially impractical and unsustainable solution".

Has participants' ethnicity played a role in terms of the degree of stigmatization they experienced by external services?

Author response: We feel that, based on our findings, it is hard for us to establish whether ethnicity has played a role in the degree of stigmatisation experiences by HSWs. However, we did find that educational attainment was commonly raised by interviewees, so have focused our results and discussion on this.

The quotes "underqualified" (HSW9) and “I think there is a bit of a culture of, you know, that [HCS] don't really see us hostel workers as professionals because they’re healthcare professionals. [HCS] go to university. You know, a lot of the hostel workers, they've never been to uni, they're not specialists in healthcare” (HSW11) make me particularly curious on what the qualifications and backgrounds of HSW are (see comment above).

Author response: To address your comment we have now explored this further in the discussion on page 11. Additionally, demographic data can be found in Table 2.

What are the (potential) reasons for dental care and mental health services being more difficult to navigate for HSWs?

Author response: More clarification regarding difficulties accessing dental and mental healthcare has been included in the results section on page 9. This includes discussing the wider NHS challenge of dentist shortages, and also challenges with mental health services, due to long waiting lists, rejected referrals and prompt discharges (Page 9).

Discussion:

Again, it would be beneficial to explain more about the background and qualification of HSWs and how these influence the perception of this group among other professionals.

Author response: We have included more clarification on this in the discussion and have also highlighted that little is known about the HSW workforce on page 11. Our demographic table (Table 2), also provides some demographic data on the background and qualifications of HSW included in our study.

Why is it particularly hard for HSWs to navigate access to dental care and mental health services?

Author response: We have now included further information regarding these challenges in the results section on page 9.

Please spell out the acronym ICS (Integrated Care Systems) as it is only used once throughout the manuscript.

Author response: We have now spelt out Integrated Care Systems and removed the acronym ICS from the paper (Page 11).

I urge the authors to add a concluding recommendation on how specific policies currently in force would have to be adapted in order to improve HSWs' healthcare navigation alongside PEH, particularly in terms of the issues that participants raised concerning GDPR.

Author response: We have now included a recommendation to address challenges regarding information sharing and GDPR in the Implications for Research and Clinical Practice section on page 12. Specifically we highlighted the use of consent form and confidentiality released, which have the potential to facilitate information sharing in line with regulation, providing they are recognised by the healthcare system (Page 12).

Reviewer 2 comments:

Thank you for providing me with the opportunity to review this study. The authors have recognised an important stakeholder group that have a valuable role in supporting PEH. Their perspectives rightly need to be considered especially as they are crucial to supporting PEH. After reading the submission I have the following comments/recommendations provided below.

Author response: Thank you for reviewing our paper. We appreciate your feedback and comments, and have incorporated these into our revised manuscript.

Strengths and limitations:

Whilst I appreciate that is requested this section is requested here, there is no mention of strengths and limitations in the discussion, therefore could this be addressed. Especially to consider strengths and limitations in relation to the wider literature.

I wonder if a limitation is that only those who would have been able to access MS teams could participate in the study, what about those unable to access MS teams for an interview, there is no evidence from the manuscript whether any other options for interviews were provided (e.g., telephone, face to face).

Author response: Thank you for pointing this out. We have now included limitations in our discussion on Page 11. Specifically, we have included the limitation you highlighted regarding the use of Microsoft Teams, as well as limitations regarding the fact this study was unfunded, meaning that participants volunteered their time to be interviewed (Page 2). We have demonstrated strengths by highlighting our novel findings in the context of the wider literature throughout our discussion on pages 11 and 12.

The authors mention that the researchers who conducted the interview were healthcare students, and this could have created response bias, response bias is not appropriate for qualitative research particularly work applying a phenomenological epistemology. You are exploring lived experiences, so saying this suggests you are thinking people withheld information or did not report accurately on their lived experiences which is unfair to your participants. I think the authors may be trying to describe positionality and the potential impact of this, but it’s unclear why this would be a limitation as authors mention an effort to build rapport was made prior to data collection. This would generally assume participants would feel comfortable sharing both positive and negative experiences…

Author response: We agree that response bias is not appropriate for our study design, and have addressed this in the Strengths and Limitations section of the paper on Page 2. Additionally, a positionality statement is now included in the Research Team, Reflexivity and Positionality section of the paper; whilst understanding the relation of this to both our epistemology and biases (Page 3).

The authors describe purposive sampling as a limitation but again this is one of the most appropriate strategies to employ when conducting qualitative research, therefore a strength as you are intending to purposefully sample a homogenous sample of people?

Author response: Thank you for highlighting this. We have now addressed this in the strengths and limitations section on Page 2.

It is not clear what the authors mean by the study sample not being representative, as this again is a term more applicable to a quantitative paradigm, from the discussion it suggests the findings of the study are transferable and reflect experiences reported by others, the findings from qualitative research do not need to be representative because they are not typically used for generalisability purposes. I would consider revising this, as it is not a limitation.

Author response: This has been revised and addressed in the Strengths and Limitations section of the paper on Page 2. The term ‘representative’ has been removed from the paper. Instead the transferability of the study has been mentioned. (Page 2)

Line 11: Consider changing hostel staff to HSW as you use HSW later on in the sentence so I would stay consistent throughout.

Author response: Thank you for pointing this out, this has now been changed to HSW (Page 3).

Research team and reflexivity:

There is little to no information about the experience of qualitative research across the team beyond the team has conducted semi-structured interviews. There is further limited information about positionality of the researchers, is this an area you were an insider/outsider to. How would this then impact data collection, the interpretation of findings, and analysis?

Author response: The experience of the whole research team has now been added and positionality of the researchers has been addressed in the Research Team, Reflexivity and Positionality section of the paper on Page 3.

Study design:

Authors have stated that they have applied a phenomenological theoretical framework, but it is unclear what the theoretical framework is, can you provide examples of constructs you coded into, if a theoretical framework was applied, did you code into this, if that is the case you are performing a deductive analysis.

Author response: This was initially worded poorly, so we have re-worded it to make it clearer. A phenomenological perspective was adopted as reflexive TA cannot be conducted under a theoretical vacuum. This can be found under the Study Design section on pages 3 and 4.

The authors have not engaged with member checking which I can agree with as there is limited evidence of its impact on credibility and trust worthiness, but as this was not used, what else did authors do to ensure findings are credible, represented participants reported experiences, and so trustworthy. As authors have used a phenomenological approach checking with participants around how their data has been interpreted would have been a good approach in this context. It would be good to add what steps you have taken to ensure findings were credible, and trustworthy.

Author response: This has now been addressed in the Data Analysis section of the paper, where we have described the prolonged data immersion and familiarisation process undertaken, as well as the iterative reviewing process of codes and themes. (Page 4)

Authors mention they did not discuss their goals or interests however this could have facilitated building a further rapport with participants, so could you add a sentence around why you chose not to do this, its not entirely clear why this choice was made.

Author response: This has now been addressed in the Research Team, Reflexivity and Positionality section of the paper. Participants were aware of the interests and background of the interviewers, this was worded poorly initially and has been reworded (Page 3).

As you have used reflexive TA, I would use the updated reference for Reflexive TA and check your steps in accordance with that see below:

Braun, V., Clarke, V.: Reflecting on reflexive thematic analysis. Qual. Res. Sport Exerc. Health 11(4), 589–597 (2019).

Braun, V., Clarke, V.: One size fits all? What counts as quality practice in (reflexive) thematic analysis? Qual. Res. Psychol. (2020).

Author response: Thank you for directing us to the 2020 revised framework. After reviewing the papers you highlighted, we have identified that this is applicable to the process we conducted. Therefore we have updated this reference on Page 4.

Please can you add an example of a coding tree, figure 1 is not sufficient.

You need to illustrate how you went from codes to sub-themes, and theme, so readers can follow the coding journey, this transparency contributes to the credibility of your analysis and transparency of it. I would consider doing this for one of your themes as an example, its not necessary to do for all.

Author response: We have now included a coding tree for transparency. Please see Figure 2.

Consider changing candidate themes, to themes as this language is more widely use and understood, you also refer to candidate level themes as just themes in the discussion, so be consistent throughout the manuscript.

Author response: We have now changed candidate themes to themes, and have ensured this is consistent throughout the manuscript (Pages 4 and 7)

Results:

There are a lot of acronyms used in the narrative of the results, this hinders the readability of the findings I would consider removing HSC, and writing this out instead.

Author response: Thank you for this suggestion. We did consider writing out HCS, however given word count constraints, and the high frequency in which we use this acronym, we feel it is more appropriate to use HCS throughout the paper.

Theme 1:

The narratives seem very descriptive, given you have taken a phenomenological approach I would expect to see more analytical quotes, that move beyond description and have more interpretation beyond what is written.

Author response: The results section has been rewritten in line with this feedback, in order to ensure it is analytical and thorough. The results section now has a deeper focus on interpretation and the hidden concepts that can be extracted from the data, dependent on the researchers subjectivity.

For example there is missing context in a lot of the narratives when describing themes, theme 1 can you explain what kinds of stigma, give context is the impact direct or indirect, Im assuming the impact is indirect, based on what hostel workers are told by PEH but it would be good to this to be clearer.

Author response: We have taken on board your feedback to improve this section so it includes more context regarding types of stigma (direct and indirect), and the impact of this stigma, for example, in terms of causing emotional distress for HSW and also increasing workload (page 7).

Note for quotes: The authors could benefit from clarifying quotes through using [] square brackets.

Author response: [] have now been used to clarify quotes, and provide context context to quotes (Pages 7-11).

For example: theme 1 quote from HSW12, what is hard to see? Are the HSW talking about a particular incident, a particular kind of stigma/assumption, a particular action? This will enable better readability for the audience too.

Author response: [] have been used to provide more context to this quote (and other quotes throughout the results section). For this quotes specifically. HSW12 was referring to their observations regarding how they are treated better than PEH by HCS. This context has been added, and can be found on page 7.

What kind of unconscious biases? Can you clarify using [].

Author response: This has now been clarified using [] and was referring to PEH being viewed as chaotic and disengaged on Page 9.

Subtheme 1b: there is scope to go beyond description in this theme, reading the narrative I'm left asking so what? You've discussed that HSW are directly impacted, how, they're frustrated but how does this interfere with their job? How do these assumptions impact engagement, does this cause problems with PEH in terms of them feeling let down? Does this power imbalance impact how PEH engage with HSW? What did participants discuss when sharing these experiences. When there are assumptions about lack of qualifications, how does this affect collaboration? Does it increase existing reluctancies by PEH who already have bad experiences?

Author response: This subtheme has been re-written and developed to include your comments and feedback. There is now further information on how these power imbalances impact HSW, for example by making them feel more distant from HCS and closer to PEH, and results in important information being missed by HCS as they don't value information shared by HSW (Page 8).

You would expect people gave examples of what this kind of power imbalance did, it would be good to explore this further because then you can make specific recommendations about roles and responsibilities, the knowledge sharing and why HSW shouldn't be disregarded.

Author response: A quote has been included to highlight how power imbalances result in an 'us versus them' feeling, since HSWs and PEH grow closer together, and become increasingly distant from HCS.

[HSW11 response when asked about how negative experiences with HCS impacts how they navigate access to healthcare] "It's not gonna impact me because I'll just build a closer relationship with my residents and get the information that I need". (Page 8)

Experiences of collaboration:

This theme contradicts the above theme, as power imbalances have been described but then there are largely positive experiences with GPs so perhaps consider bringing the GPs into the theme title.

Author response: we agree, and have now included GP in the subtheme title on Pages 8-9. The abstract, figure and discussion have been changed accordingly.

Experiences of disconnect:

Similar issues as described above, the narrative is very limited, and would benefit from further context e.g., what were the struggles navigating dental and mental healthcare services. Its difficult to make recommendations if the issues aren't clearly described. I appreciate that authors may be limited by word limits but this context is important to mention in the findings.

Author response: Clarification regarding difficulties accessing dental and mental healthcare have now been included under results on pages 9-10. This includes discussing the wider NHS challenge of dentist shortages, and also challenges with mental health services, due to long waiting lists, rejected referrals and prompt discharges (Pages 9 and 10).

Can you provide an example of the opposing beliefs and priorities.

Author response: We have now provided examples of opposing beliefs and priorities through context and a quote, which is shown below and can be found on page 9.

HSWs believe in repetition and persistence, which feels incompatible with NHS protocols that discharge patients who do not attend appointments.

[HSW5 discussing re-referring PEH to healthcare services, and the response from HCS] “They [HCS] say, ‘we offered this last time and they [PEH] didn't arrive, they're just never gonna engage with this’. We [HSWs] have an outlook of consistency and repetitiveness and try, try, try again, whereas health services just cannot do that.” (Page 9)

In reach – Can the authors describe what in-reach is please. An international readership may not be familiar with UK services therefore again for readability this will help and enable the theme to have more meaning.

Author response: In-reach has now been defined in the results on page 10. “In-reach is when HCS bring their expertise and services into hostels, directly to PEH.” (Page 10)

As you conducted data collection in the West Midlands, did HSWs mention nurse-led programmes for PEH? There is a physical and mental health nursing outreach programme in WM for PEH, which is really well received and the uptake is significant, and I wonder whether HSWs mentioned interactions with this or other similar community nursing programmes.

Bell, Lauren, et al. "Healthcare and housing provision for a UK homeless community: a qualitative service evaluation." Public Health 229 (2024): 1-6.

Author response: Thank you for sharing your paper with us. Participants did often mention specific services available in their area, however to maintain some confidentiality we did not disclose the names of teams in the paper. In our results section we have highlighted that the most common form of in-reach available was nurse-led (page 10). As a result, many of our quotes are referencing the in-reach work of nurses, however we have decided to use HCS to encompass a range of healthcare roles.

General comments: The manuscript is generally well-written and I believe this is an important study, the analysis however currently is very descriptive, which needs addressing.

Author response: Thank you for your feedback and suggestions. We have incorporated your comments to improve the manuscript, this included re-writing the results section with the hope that it is now more analytical and detailed.

I wonder if some of the findings particularly in relation to cross-collaborative/multidisciplinary team working could be theoretically grounded so more appropriate recommendations could be given. Perhaps organisational culture theories, social theories around power dynamics…

Author response: We have included some further context on organisational culture in our discussion on page 11. However, given the word count constraints of the paper, and the breadth of the suggested topics, we have had to keep this limited.

VERSION 2 - REVIEW

**REVIEWER NAME**	Schiffler, Tobias
**REVIEWER AFFILIATION**	Medical University of Vienna
**REVIEWER CONFLICT OF INTEREST**	None to declare.
**DATE REVIEW RETURNED**	10-Jun-2024

**GENERAL COMMENTS**	Thank you for the hard work you put into this review!<br><br>I only have one very minor comment left, as this comment was addressed in the point-by-point response but not in the manuscript:<br>How are Inclusion Health Groups defined according to the cited NHS framework?<br>Author response: This definition is now included in the introduction on page 2.<br>“PEH are an Inclusion Health Group under NHS England’s Inclusion Health Framework as they experience social exclusion, poor access to healthcare and multiple overlapping risk factors for poor health, including poverty, violence and complex trauma” (Page 2)<br><br>Please add this description to your manuscript.

VERSION 2 – AUTHOR RESPONSE

Reviewer: 1

Thank you for the hard work you put into this review!

I only have one very minor comment left, as this comment was addressed in the point-by-point response but not in the manuscript:

How are Inclusion Health Groups defined according to the cited NHS framework?

Author response: This definition is now included in the introduction on page 2.

“PEH are an Inclusion Health Group under NHS England’s Inclusion Health Framework as they experience social exclusion, poor access to healthcare and multiple overlapping risk factors for poor health, including poverty, violence and complex trauma” (Page 2)

Please add this description to your manuscript.

Author Response: Thank you for highlighting this omission from our manuscript, we have now ensured the above definition is included (Page 2).
